# Effects of molecularly targeted therapies on murine thymus: highly selective mTOR inhibitors induce reversible thymic involution

**DOI:** 10.1186/s40164-016-0044-3

**Published:** 2016-07-29

**Authors:** Suleiman Al-Hammadi, Saeeda Almarzooqi, Alia Albawardi, Abdul-Kader Souid

**Affiliations:** 1 Department of Pediatrics, UAE University, Al-Ain, P.O. Box 17666, Abu Dhabi, United Arab Emirates; 2 Department of Pathology, UAE University, Al-Ain, P.O. Box 17666, Abu Dhabi, United Arab Emirates

**Keywords:** Thymus, Lymphocytes, Immunosuppressants, Cellular respiration, mTOR inhibitors, Sirolimus, Everolimus, PI3K inhibitors, Calcineurin inhibitors, Sorafenib, Idelalisib

## Abstract

**Background:**

Blocking mTOR (molecular target of rapamycin) by sirolimus has been shown to suppress cellular respiration. The bearing of this impaired cellular bioenergetics on the mode-of-action of mTOR inhibitors has yet to be illustrated.

**Methods:**

This study investigated in vitro effects of several molecularly-targeted therapies on O_2_ consumption in thymic fragments from C57BL/6 mice.

**Results:**

Thymocyte respiration (µM O_2_ min^−1^ mg^−1^) was reduced by sirolimus and everolimus (*p* ≤ 0.007). In contrast, the dual PI3K (phosphatidylinositol-3-kinase)/mTOR inhibitors BEZ235, GDC0980 and GSK2126458, the highly-selective PI3 K-p110-δ inhibitor idelalisib and the calcineurin inhibitor tacrolimus had no effects on thymocyte respiration. Sirolimus was administered intraperitoneally on Days 0–3 and the thymus was then examined on Days 4 and 14. Cortex involution associated with increased cytochrome c and caspase-3 positive cells (apoptosis) were observed on Day 4; these changes were resolved on Day 14 (10 days after sirolimus treatment). On Day 4, the residual thymus (mostly medulla) had normal cellular respiration, decreased caspase activity and increased glutathione. Intraperitoneal administration of sorafenib (a multikinase inhibitor) or idelalisib had no effects on thymus size.

**Conclusion:**

Thus, the highly-selective mTOR inhibitors imposed specific effects on the thymus, manifested by suppression of cellular respiration and induction of apoptosis.

## Background

Inhibition of mTOR by the immunosuppressant sirolimus (rapamycin) has been shown to decrease cellular mitochondrial oxygen consumption (cellular respiration) in Jurkat cells [1]. Exposure of the cells to 0.1 µM sirolimus for 30 min resulted in about 20 % decrease in cellular respiration. This effect was accompanied by accumulation of intracellular lactate and other biomarkers of anaerobic metabolism [1]. In another in vitro study, inhibition of mTOR by 10 µM sirolimus lowered cellular respiration in murine heart and liver tissues by 40 % and in murine kidney tissue by 20 % [2]. In contrast, the calcineurin inhibitors tacrolimus and cyclosporine had minimum or no effects on cellular respiration in these organs [2]. These results are consistent with the known role of mTOR signaling in fluxing nutrients into the mitochondria [3, 4].

Inhibition of cellular respiration has been reported in several murine organs by the dual PI3K/mTOR inhibitors GSK2126458, BEZ235 and GDC0980 at drug concentrations ≥1 µM [5]. In contrast, the MEK inhibitor GSK1120212 (trametinib) and the multikinase inhibitors sorafenib and regorafenib had no effects on cellular respiration in these organs [5].

The effects of the PI3K P110δ inhibitor idelalisib on cellular respiration have been also studied in vitro in several murine tissues [6]. This drug is in clinical use for treatment of lymphoid malignancies, with a Boxed Warning concerning its potential lung, hepatic and intestinal toxicities [7]. Idelalisib (10 µM) significantly lowered lung cellular respiration by 27 % and liver cellular respiration by 20 %; respiration in the intestine, thymus, spleen and kidney was unaffected [6]. These results are consistent with the known expression of P110δ in epithelial lung and hepatic cells [8].

It remains to be seen whether derangements in cellular bioenergetics contribute to the immunosuppressive activity of mTOR inhibitors. This study investigated the in vitro effects of sirolimus and everolimus on O_2_ consumption in thymic fragments from C57BL/6 mice. In addition, the study examined the thymus morphology and the induction of apoptosis following systemic administration of sirolimus.

## Methods

### Reagents

Sirolimus (rapamycin), everolimus, tacrolimus (FK-506, fujimycin), idelalisib (CAL-101), BEZ235, GDC0980, GSK2126458, sorafenib and regorafenib were purchased from MedChem Express, LLC (Princeton, NJ). These compounds were dissolved in dimethyl sulfoxide (DMSO) at 5 mg/mL and stored at −20 °C. Pd(II) complex of meso-tetra-(4-sulfonatophenyl)-tetrabenzoporphyrin (Pd phosphor) was purchased from Porphyrin Products (Logan, UT); it was dissolved in dH_2_O and stored in small aliquots at −20 °C.

Monobromobimane (mBBr, m.w. 271.111) was purchased from Molecular Probes (Eugene, Oregon). mBBr (0.1 M) was dissolved in acetonitrile and stored at −20 °C. Glutathione (GSH) was dissolved in dH_2_O and stored at −80 °C; its concentration was measured by Ellman’s reagent. GS-bimane standards and sodium methane sulfonate were prepared as previously described [9].

The pancaspase inhibitor zVAD (*N*-benzyloxycarbonyl-val-ala-asp(*O*-methyl)-fluoromethylketone) was purchased from Calbiochem (La Jolla, CA); it was dissolved in DMSO at 7.4 mM and stored in small aliquots at −20 °C. The caspase-3 substrate Ac-DEVD-AMC (*N*-acetyl-asp-glu-val-asp-7-amino-4-methylcoumarin) was purchased from Axxora LLC (San Diego, CA); it was dissolved in DMSO at 2.14 mM and stored in small aliquots at −20 °C. Rabbit anti-cytochrome c antibody [(H-104): sc-7159] and rabbit anti-cleaved caspase-3 antibody were purchased from Santa Cruz Biotechnology, Inc. (Texas, USA). Glucose, HPLC-grade methanol, RPMI medium (Roswell Park Memorial Institute medium), dichloromethane, trifluoroacetic acid, methanesulfonic acid and remaining reagents were purchased from Sigma-Aldrich (St. Louis, MO).

### Mice

C57BL/6 (6–8 weeks old) mice were housed at 22 °C, 60 % humidity and 12-h light–dark cycles. They had ad libitum access to standard rodent chow and filtered water. The study was approved from the Animal Ethics Committee—College of Medicine and Health Sciences (A29-13; in vitro assessment of the effects of nephrotoxic drugs and toxins on renal cellular respiration in mice).

### Tissue collection and processing

Urethane (25 % w/v, 100 µL per 10 g, administered intraperitoneally) was used for anesthesia. The thymus was then quickly removed with a sterile scalpel and immersed in ice-cold RPMI medium saturated with 95 % O_2_: 5 % CO_2_. A fragment of the gland was immediately placed in an oxygen-measuring vial for determining the rate of cellular respiration at 37 °C [10, 11]. The cellular respiration reaction mixture contained 1.0 mL RPMI medium, 3 µM Pd phosphor and 0.5 % fat-free albumin.

**Fig. 1:**
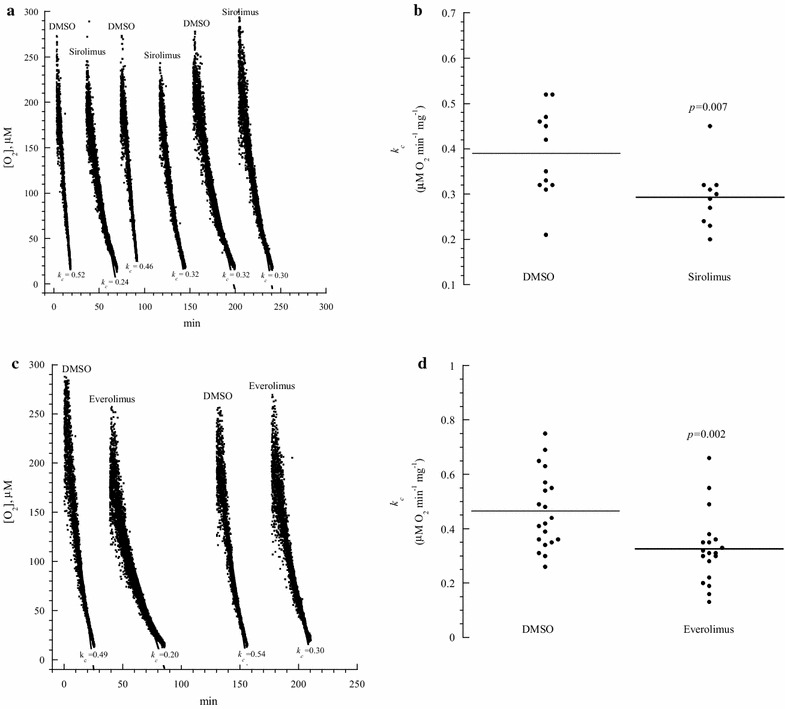
In vitro effects of sirolimus and everolimus on thymocyte respiration. **a** and **c**: One representative from 10 to 20 separate experiments. Each run represented a thymus fragment that was collected from a C57BL/6 mouse and processed immediately for measuring cellular respiration in the presence of 10 μM sirolimus (or everolimus) or 1.6 µL DMSO. The rate of respiration (*k,* μM O_2_ min^−1^) was the negative of the slope of [O_2_] vs. *t*. The values of *k*_*c*_ (μM O_2_ min^−1^ mg^−1^) are shown at the* bottom* of each run. The* lines* are linear fit. **b** and **d**: Summary of all results; the *horizontal lines* are mean

**Fig. 2:**
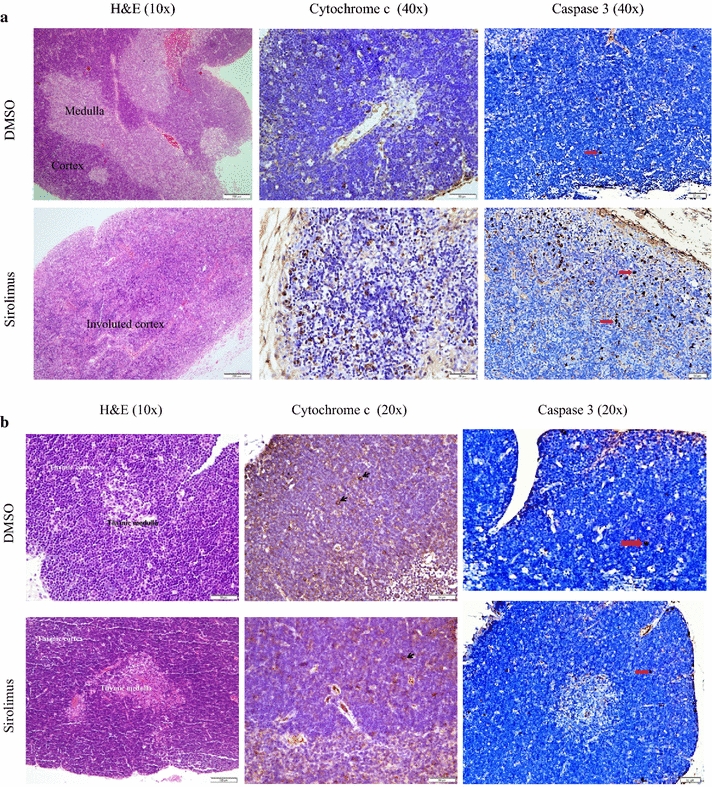
Effects of systemic sirolimus treatment on thymus histology and immunoperoxidase staining with cytochrome c and caspase 3. Mice received intraperitoneal DMSO or sirolimus on Days 0–3. Thymus fragments were then stained with H&E and immunoperoxidase with anti-cytochrome c and caspase 3 on Day 4 (*Panel* **a**) or Day 14 (*Panel* **b**).* Panel* **a** (Day 4): *DMSO (control):* Normal thymus histology demonstrating darkly staining lymphocyte rich cortex and pale staining medulla; few (<2 %) cortical lymphocytes with positive staining for cytochrome c and caspase 3 are present (*arrow*). *Sirolimus*: Thymic cortical involution with thinning of cortex and loss of the demarcation between the cortex and medulla; increased staining by cytochrome c and caspase 3 (about 20 % of the cells are positive).* Panel* **b** (Day 14): Normal thymus histology with a cortex rich in lymphocytes and a lymphocyte scant medulla; rare (<2 %) cortical lymphocytes with positive staining for cytochrome c are present (*arrow*)

**Fig. 3:**
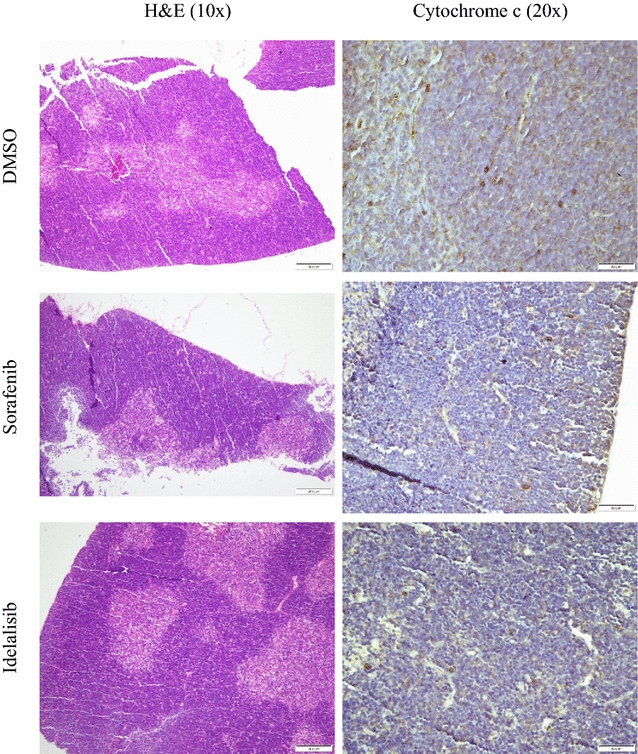
Effects of systemic sorafenib and idelalisib treatments on thymus histology and immunoperoxidase staining with cytochrome c (Day 4). Mice received intraperitoneal DMSO, sorafenib or idelalisib on Days 0–3. Thymus fragments were then stained with H&E and immunoperoxidase with anti-cytochrome c on Day 4. Normal thymus histology with a cortex rich in lymphocytes and a lymphocyte scant medulla; few (<2 %) cortical lymphocytes with positive staining for cytochrome c are present

**Fig. 4:**
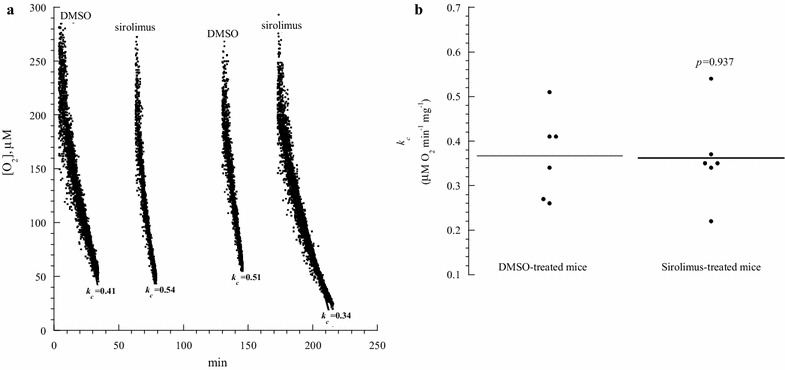
Thymocyte respiration in mice treated with sirolimus (Day 4). **a** One representative from three separate experiments. Mice received intraperitoneal DMSO or sirolimus on Days 0–3. On Day 4, thymic fragments were collected and processed immediately for measuring cellular respiration. The rate of respiration (*k,* µM O_2_ min^−1^) was the negative of the slope of [O_2_] vs. *t*. The values of *k*_*c*_ (µM O_2_ min^−1^ mg^−1^) are shown at the bottom of each run. The* lines* are linear fit. **b** Summary of all results; the *horizontal lines* are mean

**Fig. 5:**
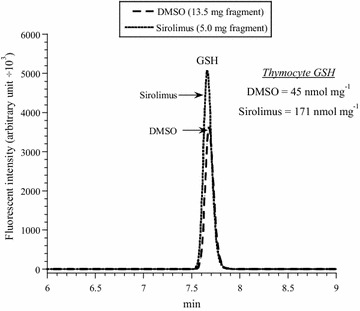
Thymocyte GSH in mice treated with sirolimus (Day 4). One representative from three triplicate experiments is shown. Mice received intraperitoneal DMSO or sirolimus on Days 0–3. On Day 4, thymus fragments were collected and processed immediately for measuring cellular GSH. The GS-bimane derivatives (retention time, *R*_t_ = 7.7 min) were separated on HPLC and analyzed by fluorescence. The values of GSH (nmol mg^−1^) are shown

**Fig. 6:**
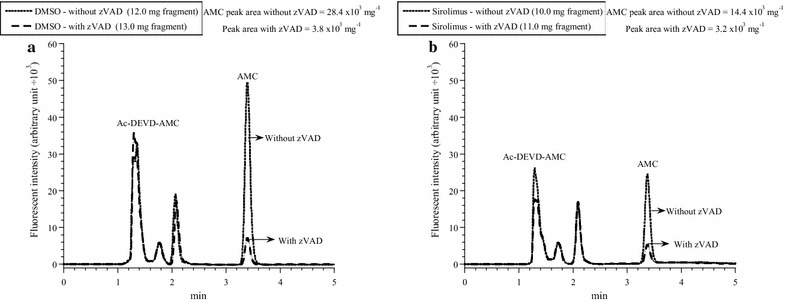
Effects of systemic sirolimus treatment on thymocyte caspase activity (Day 4). One representative from three triplicate experiments is shown. Mice received intraperitoneal DMSO (**a**) or sirolimus (**b**) on Days 0–3. On Day 4, thymic fragments were collected and incubated with the caspase-3 substrate Ac-DEVD-AMC with and without zVAD (pancaspase inhibitor) as described in “Methods” section. The tissue homogenates were separated on HPLC and analyzed by fluorescence for the released AMC moieties (reflecting intracellular caspase activity, *R*_t_ = 3.44 min*).* The values of AMC peak areas (arbitrary unit ÷ 10^3^ per mg) are shown. AMC peak areas decreased by ≥78 % in the presence of zVAD, confirming the cleavage was mediated mainly by caspases

For the in vitro studies (Fig. 1; Table 1), thymocyte respiration was measured in the presence of 1.6 µL DMSO or 10 µM study drug. For the in vivo studies (Figs. 2, 3, 4, 5, 6; Table 2), mice received intraperitoneal injections of DMSO (0.5 µL/g), sirolimus (2.5 µg/g) or other tested drugs (2.5 µg/g) from Day 0 to Day 3. The thymus was then removed on Day 4 (1 day after treatment) or Day 14 (10 days after treatment) and processed for assessment of morphological changes, immunohistochemistry (cytochrome c and caspase three positive cells), cellular respiration, cellular GSH and intracellular caspase activity.

**Table 1:** In vitro effects of the studied drugs on thymocyte respiration

	Drug concentration (µM)	*k*_*c*_ (µM O_2_ min^−1^ mg^−1^)	Inhibition (%)	*P*
Sirolimus (mTOR inhibitor)	0	0.39 ± 0.10 (12)	–	–
	10	0.29 ± 0.07 (10)	26	0.007
Everolimus (mTOR inhibitor)	0	0.46 ± 0.14 (20)	–	–
	10	0.33 ± 0.13 (19)	28	0.002
BEZ235 (PI3 K/mTOR inhibitor)	0	0.44 ± 0.13 (12)	–	–
	10	0.35 ± 0.16 (12)	16	0.045
GDC0980 (PI3 K/mTOR inhibitor)	0	0.48 ± 0.11 (12)	–	–
	10	0.40 ± 0.08 (12)	17	0.089
GSK2126458 (PI3 K/mTOR inhibitor)	0	0.53 ± 0.16 (11)	–	–
	10	0.45 ± 0.13 (11)	15	0.193
Idelalisib (P110δ inhibitor)	0	0.44 ± 0.19 (7)	–	–
	10	0.48 ± 0.14 (8)	0	0.336
Tacrolimus (Calcineurin inhibitor)	0	0.41 ± 0.12 (12)	–	–
	10	0.45 ± 0.10 (12)	0	0.755

For each run, the thymus was excised from a C57BL/6 mouse, and a fragment of the gland was immediately placed in the oxygen-measuring vial for determining the rate of cellular respiration in the presence of DMSO or designated drug

The values of *k*_*c*_ are mean ± SD. The values in parentheses are number of mice (equal to number of O_2_ runs)

**Table 2:** Thymus weight, caspase activity, and GSH level in mice treated with sirolimus

	DMSO	Sirolimus	*P*
Mouse weight on Day 0 (g)	19.8 ± 4.3 (13)	19.0 ± 4.8 (13)	0.315
Thymus weight (mg)	39.2 ± 10.2 (13)	20.7 ± 6.9 (13)	<0.001
Thymocyte respiration	0.37 ± 1.0 (6)	0.36 ± 1.0 (6)	0.937
Thymocyte GSH (nmol mg^−1^)	44 ± 14 (9)	82 ± 45 (9)	0.019
Thymocyte caspase activity*	28 ± 15 (9)	12 ± 9 (9)	0.019

The thymus was excised from C57BL/6 mice after intraperitoneal treatments with DMSO (0.5 µL/g on Days 0–3) or sirolimus (2.5 µg/g on Days 0–3). On Day 4, the weight of the entire gland was measured and fragments of the gland were processed for measuring cellular respiration, caspase activity and GSH level as described in “Methods” section

Expressed as µM O_2_ min^−1^ mg^−1^

* Expressed as AMC peak area (in arbitrary unit ÷10^3^) mg^−1^

For histology, thymic fragments were fixed in 10 % formalin and embedded in paraffin. Four-micron sections were cut and stained with hematoxylin and eosin (H&E). Additional sections were obtained on coated slides to perform cytochrome c and caspase 3 immunostains as previously described [6]. The immunostaining intensity was scored using a semi-quantitative manual method: strong (3+), moderate (2+), weak (1+) and negative (0), as previously described [10–13]. The percentage of positive cells was noted [6].

For measuring GSH, thymic fragments were incubated in the dark at 25 °C in 10 mM Tris-MSA (pH 8.0) and 1.0 mM mBBr for 15 min. The derivatization reactions were quenched with 45 µL of 70 % perchloric acid. The samples were then disrupted by homogenization and the supernatants were collected by centrifugation (16,300 g for 90 min) through a Microcentrifuge Filter (m.w. limit = 10,000 Dalton, Sigma©). The GS-bimane derivatives were separated on HPLC and measured by fluorescence. Excitation wavelength was 390 nm and emission wavelength 480 nm. Solvent A was 0.1 % (v/v) trifluoroacetic acid/dH_2_O and solvent B was methanol. The run time was 30 min, flow rate 1.0 mL/min and injection volume 5 µL. The gradient was: 0 min = 10 % B, 5 min = 30 % B, 8 min = 40 % B, 11 min = 50 % B, 14 min = 60 % B, 17 min = 70 % B, 21 min = 80 % B, 24 min 100 % B, 27 min = 10 % B, 30 min = re-inject [15].

For measuring intracellular caspase activity, thymus fragments were incubated at 37 °C in RPMI medium containing 37 µM Ac-DEVD-AMC (caspase 3 substrate) with and without 32 µM zVAD (pancaspase inhibitor) for 55 min [14]. The tissue was then disrupted by homogenization and the supernatants were collected by centrifugation (16,300 g for 90 min) through a Microcentrifuge Filter (m.w. limit = 10,000 Dalton, Sigma©). The filtrate was separated on HPLC (Shimadzu i-Series, Japan) and analyzed for the free fluorogenic AMC moiety. Excitation wavelength was 380 nm and emission wavelength 460 nm. Ultrasphere IP column (4.6 × 250 mm) was operated at 30 °C and flow rate of 1.0 mL/min. Solvent A was dH_2_O and solvent B was HPLC-grade methanol (isocratic). Run time was 30 min and injection volume was 5 µL [6]. This analytical method measures only the free thiol form of glutathione (GSH), which is derivatized with the fluorescent agent monobromobimane (mBBr). Thus, the system detects only the bimane derivative of GSH.

### Statistical analysis

Data were analyzed on SPSS statistical package (version 19), using the nonparametric (2 independent samples) Mann–Whitney test.

## Results

*In vitro effects of the molecularly*-*targeted drugs on thymocyte respiration* Figure 1a shows one representative from ten separate experiments of thymocyte mitochondrial O_2_ consumption. Each run represented a thymic fragment that was rapidly collected from a C57BL/6 mouse and immediately placed in the oxygen-measuring vial for determining the rate of cellular respiration (*k*_*c*_, in µM O_2_ min^−1^ mg^−1^) in the presence of 1.6 µL DMSO or 10 µM sirolimus. Sirolimus significantly decreased the rate of thymocyte respiration (*p* = 0.007, Fig. 1b). Similar results were observed in the presence of everolimus (*p* = 0.002, Fig. 1c, d). In contrast, BEZ235, GDC0980, GSK2126458, idelalisib and tacrolimus had minimum or no effects on thymocyte respiration (Table 1). Thus, thymocyte respiration is specifically targeted by the highly selective mTOR inhibitors (sirolimus and everolimus).

*Effects of systemic administration of sirolimus on thymus histology and cytochrome c and caspase 3 expressions* The thymus was examined on Day 4 and Day 14 after intraperitoneal administration of 0.5 µL/g DMSO or 2.5 µg/g sirolimus on Days 0 to 3. On Day 4 (1 day after the last sirolimus treatment), the sirolimus-treated mice demonstrated significant thymic cortex involution (reduction in the size of the gland, *p* = 0.004, with depletion of cortical lymphocytes). There were clusters of epithelial cells, a feature reported in thymus involution as the lymphocytes are lost. There was also an increase in cytochrome c and caspase three positive cells, reaching up to 20 % (Fig. 2a). Cytochrome c and caspase three expressions in DMSO-treated mice showed only rare (<2 %) focal positive staining in thymic cortical lymphocytes. Medullary positive cells were rare (Fig. 2a). On Day 14 (10 days after the last sirolimus treatment), the histology and cytochrome c and caspase 3 expressions in the sirolimus-treated mice were almost identical to those in the DMSO-treated mice (Fig. 2b). Thus, sirolimus induced cortical lymphocyte apoptosis and the cortex regenerated after cessation of sirolimus treatment. For comparison, intraperitoneal administration of the multikinase inhibitor sorafenib or the p110-δ inhibitor idelalisib (2.5 µg/g on Days 0 to 3) had no noticeable effects on thymus histology or cytochrome c expression (Fig. 3).

*Effects of systemic administration of sirolimus on thymocyte respiration, GSH and caspase activity* Mice received intraperitoneal injections of 0.5 µL/g DMSO or 2.5 µg/g sirolimus on Days 0 to 3. On Day 4, the weight of the entire thymus was measured and fragments of the gland were processed for measuring cellular respiration (Fig. 4, one representative from three separate experiments performed in duplicates), cellular GSH (Fig. 5, one representative from three separate experiments performed in triplicates) and intracellular caspase activity (Fig. 6, one representative from three separate experiments performed in triplicates). The results are summarized in Table 2. It is worth noting that the measurements in sirolimus-treated mice reflected the residual thymic medulla and in DMSO-treated mice the entire thymic cortex and medulla (as shown in Fig. 2). For comparison, intraperitoneal administration of idelalisib (2.5 µg/g on Days 0 to 3) had no effects on thymus weight (Table 3).

**Table 3:** Thymus weight in mice treated with idelalisib

	No treatment	DMSO	Idelalisib
Mouse weight on the day of sacrifice (g)	21.7 ± 6.4 (4)	19.6 ± 7.1 (4) *p* = 0.886	22.4 ± 6.7 (4) *p* = 0.686
Thymus weight (mg)	31.0 ± 12.0 (4)	22.5 ± 8.3 (4) *p* = 0.486	42.5 ± 15.5 (4) *p* = 0.343

Values are mean ± SD (n)

The thymus was excised from C57BL/6 mice 24 h after intraperitoneal administration of DMSO (0.5 µL/g) or idelalisib (2.5 µg/g) for 4 or 7 days

Thymic GSH (nmol mg^−1^) in mice treated with DMSO was 44 ± 14 (n = 9) and with sirolimus 82 ± 45 (n = 9), *p* = 0.019 (Table 2). For measuring thymocyte caspase activity, the cleavage of Ac-DEVD-AMC (a caspase-3 substrate analogue) was measured in the presence and absence of the pancaspase inhibitor zVAD. The AMC peak area (reflecting intracellular caspase activity, in arbitrary unit ÷ 10^3^ per mg) for mice treated with DMSO was 28 ± 15 (n = 9) and with sirolimus 12 ± 9 (n = 9), *p* = 0.019 (Table 2). AMC peak areas decreased by ≥78 % in the presence of zVAD, confirming the cleavage was mediated mainly by caspases (Fig. 6). Thus, the sirolimus treatment resulted in a significant reduction in the size of the gland (*p* < 0.001), increase in thymic GSH (*p* = 0.019), and decrease in thymic caspase activity (*p* = 0.019). The rate of thymocyte (mostly medullary tissue) respiration, however, did not change (*p* = 0.937), Table 2.

## Discussion

This study mainly examined the effects of disrupting mTOR signals on thymocyte respiration. The highly-selective mTOR inhibitors, sirolimus and everolimus, significantly lowered thymocyte respiration in vitro (Fig. 1 and Table 1). This effect was drug-specific, since the other studied molecularly-targeted agents did not exhibit similar inhibition (Table 1). This observed sirolimus-induced decrease in the rate of mitochondrial O_2_ consumption is consistent with the known role of mTOR signaling in cellular metabolism, including nutrient transport to the mitochondria [1–4].

Lower cellular respiration implies defects in any of the following processes: delivering nutrients and O_2_ to the mitochondria, oxidation of reduced metabolic fuels, passage of electrons to O_2_ and synthesis of ATP. As previously shown for idelalisib [6], mTOR inhibitors have no direct effects on mitochondria isolated from murine liver. Thus, the observed effects (Table 1 and Fig. 1) are likely due to impaired lymphocyte metabolism as a result of blocking mTOR signaling [3, 4].

It remains unknown whether BEZ235, GDC0980, GSK2126458, idelalisib and tacrolimus inhibit thymocyte respiration at doses higher than 10 µM. It is worth emphasizing that these novel molecularly targeted agents do not alter cellular bioenergetics at this relatively high concentration (10 µM). However, future studies are needed if any of these agents are proven to achieve clinical plasma levels exceeding 10 µM.

In vivo studies, on the other hand, show marked thymus involution following sirolimus treatment (Fig. 2a). This effect is due to induction of apoptosis [15], which depletes the cortical lymphocytes (Fig. 2a).

In this study, sirolimus treatment was from Day 0 to Day 3. This intervention resulted in near complete involution of the thymic cortex (Fig. 2a). Further studies are needed to investigate longer duration of treatment, perhaps using much lower drug dosing.

Further studies are needed to address the effects of administering various sirolimus dosing on the thymus. The sirolimus dose used here (2.5 µg/g daily for 4 days) has been previously used for BALB/c mice of 10 weeks of age [16]. In one study, sirolimus administration (5.0 µg/g/day) induced significant weight loss in old C57BL/6 mice [17].

This study investigated the effects of mTOR inhibitors on the thymus and compared them with those of other molecularly targeted drugs. The precise mechanism(s) by which mTOR inhibitors impair cellular bioenergetics and induce cortical lymphocyte apoptosis requires further investigations. Additional studies are also needed in various disease-bearing mice.

It is also worth noting that the measurements of cellular respiration, GSH and caspase activity are performed *immediately* after excising the thymus from the mediastinum. Separating the cortex from the medulla requires extensive tissue manipulation and will cause in vitro caspase activation, mitochondrial cell death pathway initiation and GSH oxidation. Novel methods are needed in order to perform these measurements on different histological and/or cellular compartments (e.g., thymus cortex vs. medulla).

In conclusion, the results show the highly selective mTOR inhibitors sirolimus and everolimus significantly lower thymocyte respiration in vitro. This effect is not observed with other studied molecularly targeted drugs (PI3 K/mTOR inhibitors, P110δ inhibitor and calcineurin inhibitor). Sirolimus treatment induces reversible thymic cortex involution by induction of lymphocyte apoptosis. The current results also support the use cellular respiration as a surrogate biomarker for studying molecularly targeted drugs.

## Abbreviations

mTOR: molecular target of ramamycin
PI3K: phosphatidylinositol-3-kinase
HPLC: high performance chromatography
